# The role of ‘hidden’ community volunteers in community-based health service delivery platforms: examples from sub-Saharan Africa

**DOI:** 10.3402/gha.v8.27214

**Published:** 2015-03-12

**Authors:** Natalie Leon, David Sanders, Wim Van Damme, Donela Besada, Emmanuelle Daviaud, Nicholas P. Oliphant, Rocio Berzal, John Mason, Tanya Doherty

**Affiliations:** 1 Health Systems Research Unit, South African Medical Research Council, Cape Town, South Africa; 2 School of Public Health, University of the Western Cape, Cape Town, South Africa; 3 Institute of Tropical Medicine, Antwerp, Belgium; 4 UNICEF, New York, NY, USA; 5 UNICEF, Niger Country Office, Niamey, Niger; 6 Global Community Health and Behaviour Sciences, Tulane School of Public Health and Tropical Medicine, Tulane University, New Orleans, LA, USA

**Keywords:** community-based care, community volunteers, sub-Saharan Africa, developing countries, child mortality, population health, global health, health systems

## Abstract

Community-based research on child survival in sub-Saharan Africa has focussed on the increased provision of curative health services by a formalised cadre of lay community health workers (CHWs), but we have identified a particular configuration, that deserves closer scrutiny. We identified a two-tiered CHW system, with the first tier being the lessor known or ‘hidden’ community/village level volunteers and the second tier being formal, paid CHWs, in Ethiopia, Mali, and Niger. Whilst the disease-focussed tasks of the formal CHW tier may be more amenable to classic epidemiological surveillance, we postulate that understanding the relationship between formalised CHWs and volunteer cadres, in terms of scope, location of practice and ratio to population, would be important for a comprehensive evaluation of child survival in these countries. We report on the findings from our joint qualitative and quantitative investigations, highlighting the need to recognise the ‘hidden’ contribution of volunteers. We need to better characterize the volunteers’ interaction with community-based and primary care services and to better understand ways to improve the volunteer systems with the right type of investments. This is particularly important for considering the models for scale-up of CHWs in sub-Saharan Africa.

The history of community health workers (CHWs) pre-dates the Alma Ata conference on Primary Health Care, with a mixed picture of successes and failures (1, 2). Their positive impact has been facilitated by establishing clearly defined roles and tasks, supportive supervision, targeted incentives and consistent community and policy support (2, 3). While important, we raise an additional issue that has not yet been systematically addressed in evaluations of CHW programmes in sub-Saharan Africa: the role of volunteers based in the community. Whilst we agree with Singh and Sachs (4) that CHWs are an important cornerstone of increased access to health services, particularly for children, we have identified a distinctive configuration in Ethiopia, Mali, and Niger; a two-tiered system of community health services, with a formal CHW cadre practicing mainly at health post/village clinic level and a lesser known volunteer cadre operating at community/village and household level.

The ideas for this viewpoint emanated from a recently completed evaluation of the Catalytic Initiative/Integrated Health Systems Strengthening programme (CI/IHSS) funded by the Department of Foreign Affairs, Trade and Development Canada (DFATD) through UNICEF (5). The evaluation included six countries (Ghana, Ethiopia, Malawi, Mali, Mozambique, and Niger) that received CI/IHSS funding between 2007 and 2013 to support the strengthening of community-based delivery systems, particularly integrated community case management (iCCM) (6). Quantitative, qualitative, and economic evaluation methods were used including field visits to each of the countries. Detailed country reports are available at http://www.mrc.ac.za/healthsystems/publications.htm. The field visits and qualitative material from three of the countries revealed a two-tier configuration of community-based services which we believe deserves closer scrutiny. We postulate that understanding the relationship between more formalised CHWs and these volunteer cadres, in terms of scope, location of practice, and ratio to population, would be important for a comprehensive evaluation of the impact of CHWs in countries implementing this system, particularly with regard to child survival. This analysis would further enhance our understanding of different models for scale-up of CHW services in sub-Saharan Africa.

## Volunteers: the ‘hidden’ factor in evaluating the impact of CHWs in Ethiopia, Mali, and Niger

The latest international estimates show rapid declines in under-five mortality in many sub-Saharan African countries (7). A few of the poorest countries have managed remarkable declines, including Ethiopia, Mali, and Niger (where child mortality has halved over the past 12 years) (7). Several evaluations link these improvements to declines in malnutrition, and increases in immunisation coverage, bed net use, and access to curative health services that have often been provided by a formalised cadre of CHWs (6, 8–10). The increased provision of curative health services by CHWs (often paid and linked to the health system) has been credited for its positive impact on child survival in sub-Saharan Africa (8) with relative silence on other health promotion and prevention interventions that may have contributed to increased demand for health care and improved child survival.

In Ethiopia, Mali, and Niger, the formal CHW cadre has been endorsed and promoted by governments and donors since the early 2000s. They are formally recruited, with at least primary-level schooling, are salaried and operate often out of fixed health posts situated in communities (in Niger and Ethiopia) or out of their homes (in Mali) (3, 8, 11–14). In all three countries, changes in the policy environment provided an opportunity for the CHW cadre to be formalised in order to promote access to basic health care services, and inclusion of a curative component to their child health services (see Table 1).

**Table 1:** Policy background and roles of CHWs and volunteers in Ethiopia, Mali and Niger

	Policy background	Roles of CHWs and volunteers
Ethiopia	2003: Health Extension Programme (HEP) launched which aimed to provide universal access to primary health care services (24, 25), mainly preventive, through more than 34,000 locally recruited, government-salaried mostly female health extension workers (HEWs) who receive 1 year of training. 2009: Policy change allowing HEWs to dispense antibiotics for suspected pneumonia at community level. 2011: Establishment of network of volunteers (Health Development Army), drawn from ‘model family’ households, support the HEWs by providing essential health messages to the community.	**Health extension worker (HEW) roles** Two HEWs have been placed in each health post to serve a kebele, the smallest administrative unit of about 5,000 people. HEWs spend 75% of their time on outreach activities: conducting household visits organizing communities to participate in the expansion of HEP services educating families to adopt healthy life-styles and serve as ‘model families’ in their neighbourhood HEWs focus on delivering 16 primary health care (PHC) packages of services including: family health promotion and services communicable disease prevention and control hygiene and environmental health health education and communication services Child survival strategies implemented under the HEP include: immunisation vitamin A distribution distribution of bed nets treatment of fever (suspected pneumonia) with anti-malarials community-based treatment of diarrhoea, suspected pneumonia and severe acute malnutrition promotion and support for early and exclusive breast feeding deworming child health and nutrition education **Volunteer/Health Development Army roles** training of ‘model families’ to implement health initiatives and act as role models behavioural change communication and social mobilisation: facility delivery, latrine use, iodised salt, immunisation nutrition promotion and counselling: infant and young child feeding practices
Mali	1990: Mali has had a long history of using CHWs for the distribution of medication and treatment, dating back to the 1990s with a variety of names such as Relais Communautaire, Village Pharmaceutical Agent, Guinea Worm Extractors and Nutrition Promoters. March 2009: The adoption of national strategy, termed Essential Community Care programme, or Soins Essential dans la Communauté (SEC), aimed to incorporate a range of services at the community level to address maternal and child morbidity and mortality. Two layers of community health workers were established: the Relais who would continue with promotion and prevention activities, and a new cadre, the Agents de Santé Communautaire (ASC), who would be trained and paid to provide treatment to children under five for malaria, pneumonia, and diarrhoea.	**Agents de Santé Communautaire (ASC) roles** ASCs were trained to deliver essential community-based care (curative, preventive, and promotional) as per the Essential Community Care strategy. They received 40 days training (15 days basic and 25 days in-service) at health facilities. They are then placed in villages where the community is expected to build them a structure or home to work from. Each ASC is expected to cover a population of 1,500, and this may include surrounding satellite villages. By 2013, the SEC programme trained 2,052 ASCs. ASCs were trained in the full package of iCCM which includes: diagnostic and treatment of ARI, malaria, diarrhoea, malnutrition, and essential care for new-borns. They provide supervision to the Relais. **Volunteer/Relais roles** Relais were trained to deliver preventive/promotional interventions as per the Essential Community Care strategy within Mali’s broader Child Survival Strategy. They are responsible for promoting key essential family practices for health promotion and disease prevention and their roles include home-based supportive care and referral of sick and malnourished children. After receiving 5 days of training, the Relais are provided with a kit that includes IEC materials, soap and a hand washing kit to support their health promotion activities, tools to control the quality of iodised salt, contraceptives (not including injectables), water treatment tablets, bed nets to distribute to households, and ORS and zinc to provide to children with diarrhoea, and other to support their work. One Relais is responsible for 50 households and it is estimated that approximately 16,000 Relais have been trained.
Niger	1999: The law established health post structures (Case de Sante), to increase access to basic primary health services in hard to reach areas and for a paid lay health worker cadre, known as Agents de Santé Communautaire (ASC), to staff the health posts (12). ASCs received 6 months of basic training. 2008: Policy change through the National Child Survival Strategy, make provision for ASCs to provide curative care including dispensing antibiotics for suspected pneumonia at health post level. 2008 to 2013: A total of 2,560 ASCs had received the 6-day iCCM training, to provide pneumonia, malaria and diarrhoea treatment for children under 5 years. 2008–2013: The CI/IHSS programme provided training of health workers, including volunteer-cadre, the Relais, on Key Family Practices (KFPs), including: breastfeeding, use of mosquito bed nets and ORS (Oral Rehydration Salt), hand washing. 2012: Minimum package of PHC services extended for health posts with ASCs and nurses now trained to provide care for new-borns.	**Agents de Santé Communautaire (ASC)** In 2013, there were 1997 functional health posts and 1,535, mostly male (75%) ASCs deployed (27). Some health posts combined ASCs and nurse cadres. ASCs spent the majority of their time at the health post and focussed on delivering basic PHC services including: preventive and curative care for children adults antenatal care reproductive health services (family planning) communicable disease prevention and control hygiene and environmental health promotion health education and awareness raising **Child survival strategies implemented by ASCs include** treatment of fever (suspected pneumonia) with anti-malarials treatment of diarrhoea, suspected pneumonia immunisation at health post level and support for outreach campaigns vitamin A distribution and distribution of bed nets child health promotion and nutrition support screening of acute malnutrition and treatment of Moderate Acute Malnutrition (MAM) identification and referral of children with serious illness, including severe malnutrition. **Volunteer/Relais roles** The Relais work closely with both the ASCs and the clinicians at the health centre in their catchment area, at health facility level, community/village level and household level. Demonstrating KFPs, doing health promotion and community sensitisation (e.g. help organize and prepare the community for immunisation and other outreach activities). Do home-visits with ASCs (or on behalf of ASCs), for health promotion and alerting households to the signs and symptoms of sick or malnourished children, and motivate parents to seek care. Help create community awareness and give health promotion talks. In some parts of Niger they are used to deliver the Community-Led Total Sanitation programme.

In all three countries, however, there is a first tier of exclusively volunteer community-based lay health workers, whose role is less well understood (11, 13–15). They operate semi-formally within communities, at village and household level, without necessarily requiring formal education, and with limited training. The volunteers go by different names; in Niger and Mali, they are called Relais Communautaire (or Relais) (13, 15, 16, 18), and in Ethiopia, they are members of the Health Development Army (13). They engage in health and nutrition promotion and disease prevention activities at village and household level as well as social mobilisation and referrals to CHWs and health facilities.

## Task shifting of curative care and the gap filled by volunteers

A major focus for CHWs in the Alma Ata Declaration was community involvement in prevention and promotion, but due to several factors, including the challenge of the health Millennium Development Goals, there has been a recent shift in sub-Saharan countries towards using CHWs to increase access to treatment (18). In these countries, CHWs were previously recruited (often drawing from a pool of volunteers operating at village level) and provided with basic health training to deliver various services including health promotion, immunisation, family planning, improved infant and young child feeding practices, hygiene, disease control, and curative care. With the increased momentum towards child survival goals in sub-Saharan Africa, this tier of CHWs has increasingly focussed on community treatment of malaria, pneumonia, and diarrhoea and acute malnutrition (collectively known as integrated community case management or iCCM), though evidence of the effectiveness of these programmes at scale remains inconclusive (19). There is also now a push to include essential newborn care and some maternal health interventions in the scope of CHW's practice. Donor partners have increasingly funded iCCM training, medicines, equipment, and tools for supportive supervision, alongside strengthening of supply chain and monitoring systems (20). The discourse has been one of ‘task shifting’ within primary health care, from clinic level, and formal clinicians, to CHWs at the health posts (21).

The volunteer cadres, however, have a strong focus on health promotion and disease prevention, through raising community awareness, mobilising communities, sparking community dialogue, and promoting and demonstrating essential family practices including long-lasting insecticidal nets, infant and young child feeding practices, proper hygiene, and immunisation (6, 9, 12–14, 17, 22, 23). They work closely with both CHWs at health post and clinicians at health centres in their catchment area. Volunteers may accompany CHWs on home-visits; do home-visits on behalf of CHWs or conduct health promotion talks at health facilities. They also identify sick and malnourished children, encourage parents to seek health care at the CHWs and health facilities, and provide community-based oral rehydration therapy and nutritional support (6, 16, 17, 22) (see Table 1).

The Relais in Niger and Mali and the Health Development Army members in Ethiopia usually come from and live in the community and they form the link between the community and health system, participate in village health committee structures (23) and are well appreciated in communities (6, 16, 23). In Ethiopia, it is mostly women who form the Health Development Army, whilst in Mali the Relais are mostly male. In Niger, the volunteers are often respected, influential elders, both men and women; some women are also traditional birth attendants. In Ethiopia, the Health Development Army forms part of the government's social protection programme (13) and members encourage ‘model’ families and deliver the Community-based Nutrition Program through counselling to mothers, home-visits to monitor child growth, and referral of sick or malnourished children to health posts (which can lead to life-saving therapeutic feeding) (9). In Niger and Mali, donors have encouraged the development of this volunteer cadre by providing them with training in essential family practices (6, 12, 14, 22) and community-led total sanitation (26).

While the Relais are a volunteer cadre, they also receive some incentives. These include in-kind gifts such as product crops, one-time payments or stipends during training and campaigns, and equipment for their work.

## How might volunteers make a difference? Coverage and density

Until recently, there were no official country-level numbers of volunteers, but indications are that the ratio of volunteers to the population is much higher than the ratio of formal CHWs (13, 22). In Niger, CHWs are supported by at least one Relais, with more Relais operating at village level. A 2013 census of Nigerien health posts and integrated health centres (clinics) confirmed that for the 1997 health posts surveyed, there was almost double the number of Relais to CHWs, and 5–6 Relais for each of the clinics (27). In Mali, the Relais are responsible for approximately 50 households each.

A high volunteer-to-population ratio would increase the frequency of contact with community members and thus the potential impact on behaviour change and coverage of health interventions. An evaluation of the nutrition support programme in Ethiopia conducted in 2012 concluded that volunteers most likely contributed to a drop in stunting levels and underweight children and that the high ratio of volunteers to households (around 1 volunteer to 10–15 households and about 10 volunteers to each full-time CHW) was central to this success (9). The evaluation report noted that this was a mutually supportive relationship in that the support volunteers received from CHWs was key to the effectiveness of the volunteers (9). The ratio of volunteers to population is close to the volunteer-to-child population ratio of 1:10, the WHO-recommended optimal density for effective preventive health care (3, 28). There is also now stronger evidence for a high ratio of community outreach workers-to-population for child survival, as seen in Mali, where health system strengthening interventions have combined increasing the CHW-to-population ratio, with increased community-based health promotion activities and active case-finding of sick children (29). And in India, participatory women's groups and home-visits were shown to improve maternal and newborn health (30).

## What should happen next?

Sustaining community-based health service delivery platforms will require supply-side resources (such as health posts, health personnel, including CHWs, regular supervision, drugs, and monitoring and evaluation), but also demand generation, health promotion and prevention, and community-based efforts to address the social determinants of health. The challenge remains how to optimise the potential benefits of the volunteer cadre in relation to formal CHWs, whilst retaining elements that may be crucial to their impact; the spirit of volunteerism, the community social mobilisation, the focus on household-level prevention and the density (ratio) required for sufficient community linkages to the health system. Whilst disease-focussed formal CHW tiers may be more amenable to classic epidemiological surveillance, it is important to give a fuller picture of the contribution of the volunteer cadre, whose role we think is currently underestimated. In Kenya, recently a stakeholders workshop was held focussing on CHW planning where a two-tiered CHW model was discussed, namely Community Health Extension Workers (CHEWs) and Community Health Volunteers (CHVs) cadres (31).

In the haste to scale-up formally trained and salaried CHWs in sub-Saharan Africa, let's consider what model of community-based health care we are encouraging. As evaluators and policy makers, we should aim to identify more carefully the contributions of community/village-based, volunteer, lay health workers where this cadre exists, and their interaction with formal CHWs. This is also important for balancing curative and preventative services so that efforts to scale-up one do not displace the other. Joint qualitative and quantitative investigations are needed to recognise the ‘hidden’ contribution of volunteers, to better characterize their interaction with community-based and primary care services, and to better understand ways to improve the voluntary systems.

### Summary points

1. CHWs have become increasingly formalised and are considered an important cornerstone for increasing access to basic health services, including curative care for children in sub-Saharan Africa.
2. A range of factors have been identified that affect the positive impact of CHWs, and we have identified a particular two-tier configuration of a formalised, paid, often facility-based CHW cadre and a ‘hidden’ volunteer, community/village-based cadre that have not been systematically addressed in evaluations of CHW impact.
3. Understanding the relationship between formalised CHWs and these ‘hidden’ volunteers, in terms of scope, location of practice and ratio to population, would be important for a comprehensive evaluation of the impact of CHWs in countries implementing this two-tier configuration, particularly with regard to child survival.

## References

[1] Lehmann U, Sanders D (2007). Community health workers: what do we know about them? The state of the evidence on programmes, activities, costs and impact on health outcomes of using community health workers.

[2] Hill Z, Dumbaugh M, Benton L, Kallander K, Strachan D, ten Asbroek A (2014). Supervising community health workers in low-income countries: a review of impact and implementation issues. Glob Health Action.

[3] Haines A, Sanders D, Lehmann U, Rowe AK, Lawn JE, Jan S (2007). Achieving child survival goals: potential contribution of community health workers. Lancet.

[4] Singh P, Sachs JD (2013). 1 million community health workers in sub-Saharan Africa by 2015. Lancet.

[5] Canadian International Development Agency The Catalytic Initiative to save a million lives 2007. http://www.acdi-cida.gc.ca/acdi-cida/acdi-cida.nsf/eng/NAD-1249841-JLG.

[6] Doherty T, Besada D, Zembe W, Daniels K, Kinney M, Kerber K (2014). Report on the summative external evaluation of the Integrated Health System Strengthening programme in Ethiopia, Mali, Mozambique, Ghana, Malawi and Niger.

[7] UNICEF (2013). Committing to child survival: a promise renewed Progress report: Executive summary 2013.

[8] Amouzou A, Habi O, Bensaid K, Niger Countdown Case Study Working Group (2012). Reduction in child mortality in Niger: a Countdown to 2015 country case study. Lancet.

[9] White J, Mason J (2012). Assessing the impact on child nutrition of the Ethiopia Community-based Nutrition Program.

[10] Doherty T, Daviaud E, Oliphant N, Zamasiya T (2014). Community case management of childhood illness in Africa. Do people centered delivery platforms contribute to the goal of universal access?.

[11] Oliver K, Young M, Oliphant N, Diaz T, Kim J (2012). Review of systematic challenges to the scale-up of integrated community case management: emerging lessons & recommendations from the Catalytic Initiative (CI/IHSS).

[12] Leon N, Besada D, Sanders D, Daviaud E, Kerber K, Rohde S (2014). Report on the summative external evaluation of the Catalytic Initiative (CI)/Integrated Health Systems Strengthening (IHSS) Programme in Niger.

[13] Doherty T, Loveday M, Nsibande D, Daniels K, Daviaud E, Kate Kerber K (2014). Report on the summative external evaluation of the Catalytic Initiative (CI)/Integrated Health Systems Strengthening (IHSS) Programme in Ethiopia.

[14] Besada D, Sarah Rohde S, Emmanuelle Daviaud E, Kerber K, Doherty T (2014). Report on the summative external evaluation of the Catalytic Initiative (CI)/ Integrated Health Systems Strengthening (IHSS) Programme in Mali.

[15] Hercot D, Doherty T, Hongoro C, Van Damme W, Sanders D (2013). Reduction in child mortality in Niger. Lancet.

[16] Bedford J (2012). Qualitative study to identify solutions to local barriers to care-seeking and treatment for diarrhoea, malaria and pneumonia in select high burden countries: report on findings from Niger.

[17] Taylor H (2010). Mid term review of progress: Catalytic Initiative Mission Report.

[18] Bennett S, George A, Rodriguez D, Shearer J, Diallo B, Konate M (2014). Policy challenges facing integrated community case management in Sub-Saharan Africa. Trop Med Int Health.

[19] Druetz T, Siekmans K, Goossens S, Ridde V, Haddad S (2015). The community case management of pneumonia in Africa: a review of the evidence. Health Policy Plan.

[20] Rasanathan K, Muniz M, Bakshi S, Kumar M, Solano A, Kariuki W (2014). Community case management of childhood illness in sub-Saharan Africa – findings from a cross-sectional survey on policy and implementation. J Glob Health.

[21] Lehmann U, Van Damme W, Barten F, Sanders D (2009). Task shifting: the answer to the human resources crisis in Africa?. Hum Resour Health.

[22] UNICEF Niger (2014). Les Pratiques Familiales Essentielles Un modèle de communication pour le changement social et de comportement en faveur de la survie de l'enfant.

[23] Ayisi PR (2013). Les relais communautaires luttent fermement contre la mortalité de l'enfant.

[24] Wakabi W (2008). Extension workers drive Ethiopia's primary health care. Lancet.

[25] Koblinsky M, Tain F, Gaym A, Karim A, Carnell M, Tesfaye S (2010). Responding to the maternal health care challenge: the Ethiopian Health Extension Program. Ethiop J Health Dev.

[26] UNICEF (2011). Roll-out evaluation of “community led total sanitation” in West and Central Africa: final report.

[27] République du Niger Ministère de la Santé Publique, UNICEF, Institut National de la Statistique Niger (2013). Évaluation de la Mise en Oeuvre de l'Initiative Catalytique dans les Cases de Santé et les Centres de Santé Intégrés.

[28] World Health Organization (2012). Essential nutrition actions: improving maternal-newborn-infant and young child health and nutrition.

[29] Johnson AD, Thomson DR, Atwood S, Alley I, Beckerman JL, Kone I (2013). Assessing early access to care and child survival during a health system strengthening intervention in Mali: a repeated cross sectional survey. PLoS One.

[30] Prost A, Colbourn T, Seward N, Azad K, Coomarasamy A, Copas A (2013). Women's groups practising participatory learning and action to improve maternal and newborn health in low-resource settings: a systematic review and meta-analysis. Lancet.

[31] One Million Community Health Workers Campaign Update from the field: Kenya hosts stakeholders meeting on CHW planning 2014. http://1millionhealthworkers.org/2014/06/10/update-from-the-field-kenya-hosts-stakeholders-meeting-on-chw-planning/.

